# Granulocyte Colony-Stimulating Factor and Its Potential Application for Skeletal Muscle Repair and Regeneration

**DOI:** 10.1155/2017/7517350

**Published:** 2017-12-07

**Authors:** Craig R. Wright, Alister C. Ward, Aaron P. Russell

**Affiliations:** ^1^Institute for Physical Activity and Nutrition (IPAN), School of Exercise and Nutrition Sciences, Deakin University, Geelong, VIC, Australia; ^2^Centre for Molecular and Medical Research, School of Medicine, Deakin University, Waurn Ponds, VIC, Australia

## Abstract

Granulocyte colony-stimulating factor (G-CSF) was originally discovered in the context of hematopoiesis. However, the identification of the G-CSF receptor (G-CSFR) being expressed outside the hematopoietic system has revealed wider roles for G-CSF, particularly in tissue repair and regeneration. Skeletal muscle damage, including that following strenuous exercise, induces an elevation in plasma G-CSF, implicating it as a potential mediator of skeletal muscle repair. This has been supported by preclinical studies and clinical trials investigating G-CSF as a potential therapeutic agent in relevant disease states. This review focuses on the growing literature associated with G-CSF and G-CSFR in skeletal muscle under healthy and disease conditions and highlights the current controversies.

## 1. Granulocyte Colony-Stimulating Factor (G-CSF)

Granulocyte colony-stimulating factor (G-CSF) is a glycoprotein first recognized for its ability to facilitate the formation of neutrophilic granulocyte colonies in soft agar from bone marrow cells [1, 2]. Endogenous production of G-CSF is largely stimulated by infection and tissue damage. Although numerous cell types can produce G-CSF, it is primarily induced by immune cells such as macrophages as well as the endothelium [3, 4] and binds to a cognate receptor. Marketed as Neupogen® (filgrastim) (AMGEN®), recombinant G-CSF was introduced into phase I clinical trials in the mid-1980s, to restore neutrophil numbers in patients receiving chemotherapy [5]. The primary symptom for G-CSF administration is immunodeficiency, particularly neutropenia. It is used to treat severe chronic neutropenia (SCN) and neutrophil deficiencies associated with leukemia and other hematopoietic disorders [6–9], as well as neutropenia induced by chemotherapy [10–12], following bone marrow ablation prior to transplantation [13, 14], or neutrophil deficiencies caused by antiviral medications [15]. Recombinant G-CSF is administered subcutaneously or intravenously with maximal serum concentrations around 40–50 ng/ml being reached after 2–8 hours [16]. At low doses, G-CSF is able to mobilize peripheral blood progenitor cells, which has seen its application used for blood banking procedures that have largely removed the need for bone marrow transplantation [17, 18].

## 2. Granulocyte Colony-Stimulating Factor Receptor (G-CSFR) and Intracellular Signalling Pathways

**Figure 1:**
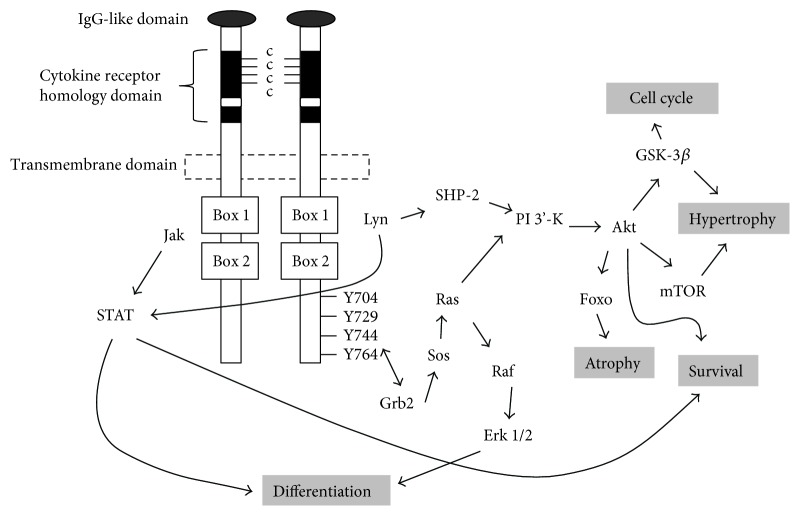
Schematic representation of the G-CSF and intracellular signalling pathways.

G-CSF's biological activity is mediated through a specific cognate receptor (G-CSFR) that belongs to the class I cytokine receptor superfamily [3, 19, 20]. The G-CSFR has a large glycosylated extracellular region that includes an N-terminal immunoglobulin- (Ig-) like domain, a cytokine receptor homology (CRH) domain, and three fibronectin type III (FBN) domains [21] (Figure 1). The CRH is an approximately 200 amino acid sequence that consists of four conserved cysteine residues and a Trp-Ser-X-Trp-Ser (WSXWS) motif, a hallmark of the class I cytokine receptors [22]. The CRH domain is involved in ligand recognition that is essential for the dimerization of two or more receptor chains and crucial for signal transduction [23]. The Ig and FBN domains contribute to receptor stability. The extracellular domain is separated from the intracellular domain by a short transmembrane sequence. Intracellularly, the membrane-proximal domain contains conserved Box 1 and Box 2 motifs and a tyrosine residue (Y704) important for proliferative signalling [24] (Figure 1). The distal domain contains a less conserved Box 3 motif associated with receptor trafficking [25] and three additional tyrosine residues (Y729, Y744, and Y764) important for proliferation, differentiation, and survival [24, 26] (Figure 1). Studies conducted in neutrophils show that only a few receptors need to be occupied by G-CSF to elicit a maximal biological response [27, 28].

Ligation of G-CSF causes conformational changes in G-CSFR that activates members of the Janus kinase family (JAK1, JAK2, and TYK2), cytoplasmic tyrosine kinases associated with Box 1 [24] (Figure 1). Activated JAKs subsequently phosphorylate the G-CSFR complex, creating docking sites for a variety of signalling molecules. This includes members of the STAT family of transcription factors [29, 30], particularly STAT3 and to a lesser extent STAT1 and STAT5 [31], which homo- or heterodimerize and translocate to the nucleus where they bind DNA and activate the transcription of responsive genes [32]. Also recruited are members of the Src family of tyrosine kinases, particularly Lyn and Hck, which activate phosphatidylinositol-3-kinase (PI3K) [33, 34] that in turn phosphorylates and activates Akt [35, 36] (Figure 1). Akt, a serine/threonine protein kinase, plays a role in many cellular processes such as glucose metabolism, cell survival, cell proliferation, and protein synthesis via numerous downstream targets [37–40] and is a major signalling pathway in skeletal muscle. Recruitment of a Grb2/Shc complex to Y704 and Y764 leads to activation of the MAPK family members, ERK1 and ERK/2, via the RAS/RAF/MEK pathway [41] (Figure 1). ERK1/2 translocates to the nucleus and activates a wide range of transcription factors and phosphorylates the protein kinase p90 ribosomal S6 kinase (p90RSK) to initiate protein synthesis [42].

These signalling pathways appear conserved in many tissues now postulated to express a functional G-CSFR. For example, PI3K/Akt pathways are activated by G-CSF in cultured neurons [43]. JAK/STAT signalling pathways [44] and PI3K pathways [45] are activated following myocardial infarction and/or heart failure, and similarly, JAK/STAT signalling pathways are activated in cultured cardiomyocytes [44]. In skeletal muscle, JAK/STAT and PI3K/Akt pathways are thought to be activated by G-CSF rodent models of muscle damage [46] and in cultured muscle cells *in vitro* [47]. Therefore, not surprisingly G-CSF treatment is suggested as a potential therapeutic target for a wide range of diseases outside the hematopoietic system.

## 3. G-CSF/G-CSFR Outside the Hematopoietic System

Expression of the G-CSFR is predominantly in cells of the hematopoietic system with the highest expression in neutrophils [48, 49]. G-CSF is well known as a hematopoietic cytokine that stimulates the proliferation, differentiation, and function of myeloid progenitors and mobilization of hematopoietic stem and progenitor cells [48, 49]. In recent years, G-CSFR expression has been identified on cells outside the hematopoietic system [24, 48], indicating a much wider role. G-CSFR is expressed on glial cells during neural development [50], and G-CSF has shown therapeutic benefits in neural tissue [43, 51, 52]. Rat cortical neural cells were protected against apoptotic death *in vitro* following G-CSF treatment [43]. G-CSF attenuated apoptotic death and improved the functional outcome in experimental models of spinal cord injury [53, 54] and motor function and life expectancy in the SOD1 (G93A) transgenic mouse, a rodent model for amyotrophic lateral sclerosis (ALS) [55]. G-CSF treatment also improved memory in rodent models of Alzheimer's disease [56], while contributing to regenerating following ischemic stroke [57, 58]. Similarly, the G-CSFR has been identified on cardiomyocytes and G-CSF stimulates cardiac myocyte proliferation during mouse development [59]. Improvements in cardiac function and cardiomyocyte survival following an experimental myocardial infarct in rodents were observed with G-CSF treatment [44].

Numerous clinical trials have been completed in patients following acute myocardial infarction [60]. While early studies showed significant improvements in left ventricular end-diastolic volume and ejection volume [61, 62], others have not [63, 64]. Meta-analysis was unable to elicit a clear answer as to the benefits of G-CSF following cardiac damage [65] but the beneficial effects of G-CSF continue to dominate the literature. Similarly, a small clinical trial demonstrated improved neurological function in stroke patients when administered G-CSF [57], while stage IIa clinical trials established that G-CSF was safe at high doses for stroke victims [66]. However, a larger stage IIb clinical trial concluded that G-CSF did not impart positive effects on stroke victims when administered intravenously ≤9 hours poststroke onset [67].

## 4. The Role of G-CSF in Skeletal Muscle

G-CSF is a well-established and well-tolerated therapeutic drug, with a growing dogma that it is beneficial in the context of repair and regeneration outside the hematopoietic system. Recently, there is growing evidence for G-CSF treatment of skeletal muscle myopathies. However, conflicting results suggest that there is still much to understand before G-CSF can be considered as a therapeutic drug in the context skeletal muscle.

Muscle injury, including that caused by strenuous exercise, is associated with an increase in plasma G-CSF. For example, maximal treadmill exercise in elite winter-sport athletes, marathon running, concentric and eccentric endurance treadmill running, and moderate and intense resistance exercise all increase circulating G-CSF levels immediately postexercise [68, 69]. It has been postulated that the elevated G-CSF levels following exercise play a role in neutrophil mobilization and delays exercise-induced neutrophil apoptosis, which is important for activating the innate immune response to exercise [68, 69]. It may also act to elevate progenitor cell mobilization, which would serve to further enhance this effect. Certainly, systemic G-CSF levels are associated with progenitor cell mobilization following endurance, resistance, and eccentric exercise modalities [70].

Mice lacking the G-CSFR (G-CSFR^−/−^) are neutropenic but otherwise develop normally and are indistinguishable from their littermates [47, 71]. However, there is a suggestion that G-CSF is fundamental to muscle growth and development as the G-CSFR^−/−^ mice have smaller muscles than their wild-type littermates with the *rectus femoris* muscle appearing to have a smaller diameter [47]. This however is controversial, as no differences in cross-sectional area were observed [68]. G-CSF/G-CSFR being fundamental to growth and development is strengthened by the observation that muscle cells *in vitro* produce G-CSF in response to stretch-induced damage [72] and following inflammatory treatments such as long-chain free fatty acids [73] and lipopolysaccharide (LPS) treatment [74]. Furthermore, in mdx mice where constant degeneration and regeneration occurs, plasma levels are elevated, while local muscle G-CSF is reduced [75]. Since the G-CSF ligand/receptor binding causes internalization and degradation of the complex, it may be postulated that elevated G-CSF is providing protective signals and G-CSF administration may facilitate muscle regeneration and remodelling and/or influence substrate utilization leading to better functional outcome.

Various rodent models have been used to explore G-CSF as a therapeutic treatment for muscle regeneration. For example, G-CSF administration improves recovery after muscle crush injury, significantly increasing muscle strength in male Wistar rats [76]. This was associated with moderately decreased cell apoptosis, increased numbers of regenerating fibres, and increased satellite cell activation. Similarly, mice injected with snake venom to cause skeletal muscle necrosis had increased rates of regeneration and activation of known anabolic signalling pathways, such as Akt, in skeletal muscle following G-CSF treatment [46]. Improved muscle regeneration and increases in survival rates are observed with exogenous G-CSF treatment in a mouse model of muscular dystrophy [77, 78], while the rodent model of amyotrophic lateral sclerosis (ALS) had improved motor function and 55% larger muscle fibres following G-CSF treatment [55]. In future studies treating rodent models of ALS with pegfilgrastim, a long-lasting form of G-CSF attenuated inflammation and increased survival rates [79]. Therefore, exogenous G-CSF treatment may be beneficial for muscle when concentrations are elevated above physiological levels to around 40–50 ng/ml [16]. This is in contrast to the modest peak concentration physiological concentrations seen after exercise [70, 80].

In more recent human clinical trials, several studies have used G-CSF as a treatment for neuromuscular disease with promising results for muscle-related functional outcomes. Specifically, Sakuma et al. [81] and Yamazaki et al. [82] demonstrated improved neurological function after treatment with 10 *μ*g/kg G-CSF in subjects with thoracic myelopathy. This is in line with this group's previous work [83, 84] where improvements in motor function following spinal cord injuries in rodents were observed. Kato et al. [85] demonstrated reduced pain in patients with compression myelopathy. Furthermore, improved functional outcomes and independence after treatment with low-dose G-CSF were observed in a single patient with a cervical spinal injury resulting in tetraplegia [86]. Finally, improved upper limb muscle strength and reduced lower limb spasticity were observed in a patient with kyphoscoliosis [87]. It is promising to consider that G-CSF may actually be affecting the skeletal muscle and thus leading to functional improvements. But given these, disease conditions have significant involvement of neuronal pathways, and G-CSF is a known neuroprotective drug; it is also plausible that G-CSF acts on the nerves without directly affecting the muscle tissue.

### 4.1. G-CSF Signalling in Skeletal Muscle

In 2009, Naito et al. [46] used a snake venom method to induce muscle damage. Three days prior, and for 5 days following the snake venom, G-CSF was administered. Increased muscle regeneration was observed by an increase in myogenic satellite cells. Interestingly, this study also demonstrated that the Akt/GSK-3*β* signalling pathways were activated, alluding to the possibility of muscle regeneration being facilitated by intracellular signalling pathways of the G-CSFR in skeletal muscle. In 2011, bone marrow crossover transplants with G-CSFR^−/−^ mice demonstrated that bone marrow cells did not contribute to G-CSF-mediated muscle regeneration [47], suggesting a direct effect of G-CSF on skeletal muscle tissue. These studies suggested that G-CSF acts directly via its receptor in skeletal muscle and activated the downstream signalling pathways important for skeletal muscle growth and development. This prompted investigations into the possibility of a functional G-CSFR in satellite cells and mature skeletal muscle.

One study demonstrated expression of G-CSFR in mouse C2C12 myoblasts by Western blot and immunohistochemistry, with decreased levels during differentiation [47]. However, the specificity of the antibody used has been called into question by others [88]. Using RT-PCR, followed by sequencing of the PCR product, we identified the expression of G-CSFR mRNA in myoblasts and differentiated myotubes and mature muscle of human and murine origin [75]. Furthermore, we used Western blotting techniques with appropriate positive and negative controls, to confirm the presence of multiple glycosylated forms of G-CSFR protein [75] and observations consistent with studies in hematopoietic cells [89]. Therefore, we conclude that the G-CSFR is expressed in skeletal muscle.

It is important to address whether G-CSF does in fact ligate with the G-CSFR and activate intracellular signalling pathways for the G-CSFR in skeletal muscle. Known G-CSF signalling pathways such Jak/STAT, PI3K/Akt, and mitogen-activated protein kinases (MAPK) signalling pathways are known to be important for skeletal muscle. For example, STAT3, the most widely studied G-CSF signalling pathway, has been implicated in C2C12 myoblast proliferation [90, 91] and in the regeneration of rodent skeletal muscle *in vivo* [92]. Furthermore, STAT3 signalling via JAK1 prevented premature differentiation of C2C12 myoblasts [93], while STAT3 signalling via JAK2 positively regulated C2C12 differentiation [94]. Therefore, G-CSF's role in muscle cell proliferation versus differentiation could differ depending on the Jak activated. For example, chemical inhibition of Jak2 downregulates the transcription factors myoD and MEF2, and target knockdown of Jak2 by siRNA leads to downregulated myoD and MEF2 target gene transcription [94]. In contrast, siRNA-targeted knockdown of Jak1 increased myoD and MEF2 as well as MEF2 target genes [93]. Interestingly, the few studies that have conducted signalling experiments in muscle cells when treated with G-CSF have not measured Jak activation rather have focused their attention on downstream targets, predominantly STAT3 signalling.

Downstream of Jak-STAT signalling, Akt is activated by a distinct region of the G-CSFR upon G-CSF ligation in hematopoietic cells [95], and Akt is one of the most widely studied protein kinases in skeletal muscle biology. Expression of constitutively active Akt1 in mouse skeletal muscle increased myofibre hypertrophy and muscle mass [37, 96], whereas inhibition of Akt resulted in muscle atrophy [97, 98]. Moreover, homozygous dominant-negative Akt1 mice exhibit growth retardation during development, with significantly reduced body mass and a reduced lifespan [97]. For muscle cells, Akt activation via Jak2-STAT3-PI3K by G-CSF would presumably increase proliferation. Similarly, Erk1/2 activation by G-CSF leads to increased proliferation of the leukemia cell line AML-193 via a Jak2-dependent pathway [99]. In mouse myoblasts, ERK signalling positively regulates proliferation as inhibition of ERK2 blocks the G1 to S phase transition promoting differentiation [100]. Therefore, G-CSF activation of Jak2-STAT3 could activate ERK signalling leading to muscle cell proliferation.

**Figure 2:**
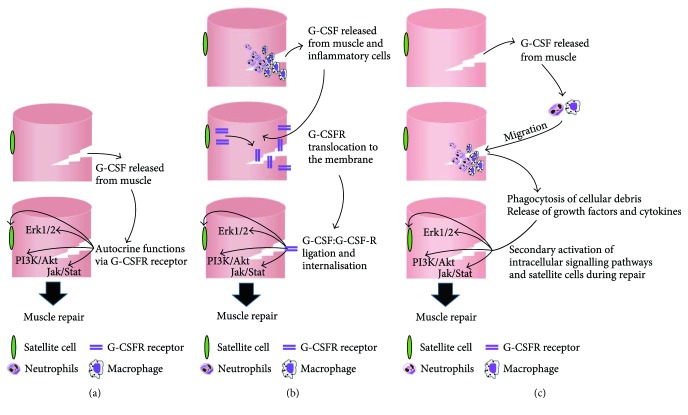
Schematic representation for the plausible mechanisms by which G-CSF aids in muscle repair.

Unfortunately, the direct action of G-CSF on skeletal muscle cells and its signalling pathways remains equivocal (Figure 1). Conflicting evidence exists in C2C12 myoblasts and myotubes with one study showing that G-CSF increases myoblast proliferation and activates STAT3, Akt, and Erk1/2 [47] which supports the notion that G-CSF has a direct effect on skeletal muscle cells (Figure 2(a)). In contrast, we observed no change in proliferation of C2C12 myoblasts with G-CSF concentrations between 400 pg/ml–100 ng/ml [75], with changes in phosphorylation of STAT3, Akt, and Erk1/2 attributed to media replenishment and not to the effect of G-CSF [75]. The concentrations used by Hara et al. [47] (<375 pg/ml) were significantly lower than those of Wright et al. [75] (400 pg/ml–100 ng/ml), and similarly much lower than the dose used in cell lines with a high expression of the G-CSFR [44, 101, 102]. Furthermore, the results produced by Hara et al. [47] are consistent with media changes observed in Wright et al. [75]. Unfortunately the methods used by Hara et al. [47] are ambiguous in that it is not clear whether the G-CSF was administered with or without fresh media, and no time point controls were presented. Therefore, these results should be interpreted with caution and it is likely that G-CSF does not activate these signalling pathways in healthy muscle cell in vivo. A null effect of G-CSF despite a functional receptor is consistent with M-CSF where despite the presence of the receptor in skeletal muscle cells, M-CSF has failed to elicit a direct biological effect [103]. Therefore, healthy cells may not respond to G-CSF treatment and cells may need to be under stress for G-CSFR to translocate to the cell surface (Figure 2(b)). This is supported by our studies in C2C12 myotubes that have shown G-CSF treatment can augment LPS-mediated IL-6 production [74] and partially alleviate the dexamethasone-induced catabolic environment [104], with no effect on the non-LPS treated cells. Therefore, G-CSF/G-CSFR signalling may in fact require an inflammatory or catabolic state in skeletal muscle to be functional. Interestingly, IL-6^−/−^ macrophages produce less G-CSF [105]. IL-6^−/−^ macrophages are associated with a decrease in myoblast proliferation and muscle regeneration *in vivo* and it is intriguing to suggest the reduced G-CSF production is contributing to this response. A third possibility is that G-CSF does not directly stimulate muscle cells. Certainly, all the rodent preclinical and human clinical trials showing improved function with G-CSF treatment are inflammatory and/or catabolic in nature and it is plausible that G-CSF influences inflammatory cells known to contribute to the repair process (Figure 2(c)).

## 5. Conclusion and Future Directions

The application of exogenous G-CSF treatment related to skeletal muscle has recently been explored, and a growing number of studies have demonstrated beneficial effects. However, the exact role of G-CSF/G-CSFR in skeletal muscle remains unclear and future studies are needed. Indeed, whether skeletal muscle cells express a functional G-CSF-R remains controversial, in part due to specificity concerns of available antibodies. Similarly, it remains unknown if the signalling pathways are activated directly in skeletal muscle due to differing results obtained from cell culture models. Therefore, the question remains as to whether G-CSF acts directly on damaged muscle cells to improve muscle health or acts on other cells such as those of hematopoietic origin, endothelial cells, and/or neuronal cells to modulate the microenvironment to favor skeletal muscle regeneration. There is a need to unequivocally determine if the G-CSF receptor is expressed in skeletal muscle and whether ligation occurs. One possibility is to perform an immunoprecipitation assay; a technique used to determine protein-protein interaction [106]. Following this, muscle-specific knockdown of the G-CSFR should be considered to determine if G-CSFR treatment does directly influence skeletal muscle. This could be achieved through a muscle-specific inducible Cre/lox strain [107] or through the more cost-effective zebrafish using CRISPR technologies [108]. More simply, to elucidate the signalling pathways in skeletal muscle, overexpression of downstream signalling targets such as Jak1 and Jak2 may augment G-CSF biological activity in skeletal muscle cells in vitro and provide conclusive evidence that G-CSF activated G-CSFR signalling pathways in skeletal muscle.

While there are promising results for the use of G-CSF to treat skeletal muscle myopathies, G-CSF has failed to elicit beneficial effects in large clinical trials of cardiomyopathies and stroke victims. This is despite promising results from *in vitro* and rodent models. Skeletal muscle may be similar, in that the early promising signs from cell culture and rodent models may not translate to G-CSF being a readily available therapeutic drug for skeletal muscle. Given the current inconsistencies in muscle cell culture signalling and the cross-reactivity of the G-CSFR antibodies, we may need more evidence before G-CSF is considered as a therapeutic treatment for muscle-related diseases.
